# Parents of Children With Type 1 Diabetes Experienced More Parent-Specific Distress Than Parents of Adolescents in China

**DOI:** 10.1155/pedi/5210513

**Published:** 2025-06-20

**Authors:** Huimei Zhao, Yun Chen, Yuwen Gao, Jie Zhong, Jiaxin Luo, Yuting Xie, Jia Guo

**Affiliations:** ^1^Xiangya School of Nursing, Central South University, Changsha, Hunan, China; ^2^School of Nursing, Xinjiang Medical University, Xinjiang Uygur Autonomous Region, Urumqi, China; ^3^School of Nursing, Li Ka Shing Faculty of Medicine, The University of Hong Kong, Hong Kong, China; ^4^National Clinical Research Center for Metabolic Diseases, Key Laboratory of Diabetes Immunology (Central South University), Ministry of Education, Department of Metabolism and Endocrinology, The Second Xiangya Hospital of Central South University, Changsha, Hunan, China

**Keywords:** adolescents, children, diabetes-specific distress, parents, type 1 diabetes

## Abstract

**Background:** Distress is one of the most common negative emotions in parents of children and adolescents diagnosed with type 1 diabetes (T1D). Because of the differences in the developmental stages between children and adolescents with T1D and their subsequent diabetes management needs, their parents may experience different levels of distress. This study aimed to compare diabetes-specific distress between parents of children with T1D and parents of adolescents with T1D in China and explore the associated factors.

**Methods:** A cross-sectional design was used. Parents of children (aged 8–12 years) and adolescents (aged 13–18 years) diagnosed with T1D for >6 months were recruited online via social media. Using established online questionnaires, data were collected on sociodemographic and T1D-related characteristics, diabetes-specific distress, anxiety symptoms, perceived stress, depressive symptoms, and parent–child conflict. Hierarchical linear regression was conducted to explore the potential factors associated with parental diabetes-specific distress.

**Results:** The final sample included 365 parents of children with T1D and 268 parents of adolescents with T1D. Notably, the parents of children with T1D exhibited a higher level of diabetes-specific distress compared to the parents of adolescents with T1D (*p*  < 0.001). Among the parents of children with T1D, higher parental diabetes-specific distress was associated with fathers with 9 years or less of education, higher annual family income (≥US$2857), higher levels of parental anxiety symptoms and perceived stress, and children's episodes of frequent hypoglycemia in the past 6 months (*F* = 8.497, *p*  < 0.001, *R*^2^ = 0.433, adjusted *R*^2^ = 0.382). Among the parents of adolescents with T1D, higher parental diabetes-specific distress was associated with fathers with 9 years or less of education and higher levels of parental anxiety symptoms and perceived stress (*F* = 4.955, *p*  < 0.001, *R*^2^ = 0.385, adjusted *R*^2^ = 0.308).

**Conclusions:** The parents of children with T1D experienced higher levels of diabetes-specific distress than the parents of adolescents with T1D in China. Fathers with 9 years or less of education and parents with more anxiety and perceived stress were particularly affected in both groups; thus, interventions are warranted.

## 1. Introduction

Type 1 diabetes (T1D) is a disease that requires lifelong insulin therapy [1]. Its prevalence is increasing worldwide, especially in children and adolescents [2]. Requisite intensive insulin therapy, regular monitoring of blood glucose levels, and adherence to a healthy lifestyle are imperative for individuals diagnosed with T1D [3]. The management of diabetes in children and adolescents, unlike that in adults, necessitates patients' reliance on their parents, either entirely or partially [4]. Parents of children/adolescents with T1D help deal with their offspring's daily diabetes-related tasks, face financial challenges, worry about their offspring's limited understanding of diabetes care [5], and are uncertain about autonomous management [6], blood glucose fluctuations, and diabetes complications in their offspring [7].

Not surprisingly, psychological issues among the parents of children and adolescents with T1D are common. A systematic review of 2012 parents found that the prevalence of psychological distress in this group ranged from 10% to 74% [8]. Notably, parents report high levels of diabetes-specific distress related to caring for a child with T1D [9]. Diabetes-specific distress can refer to the negative emotions experienced by parents or guardians of offspring with T1D [10]. Parental diabetes-specific distress results in poorer mental health and problematic behaviors in children [8], which is an even stronger predictor of children's glycemic levels than children's diabetes distress and insulin pump use [11, 12]. Therefore, it is important to develop targeted interventions to reduce parental diabetes-related distress.

Previous studies have revealed that parental diabetes-specific distress is correlated with lower parent's education levels [13], severe hypoglycemia, authoritarian parenting, less emotional support [14], and more family conflict [5, 15]. Furthermore, differences in shared T1D management responsibilities at different developmental stages of children and adolescents are likely to influence parental diabetes-specific distress [4, 16]. For instance, for children with T1D, their parents are primarily responsible for their dietary choices and daily care; adolescents with T1D begin to develop more independence in these areas [4]. Parents of adolescents with T1D need to provide more oversight to prevent the deterioration of glycemic control from insulin resistance [17].

In China, the prevalence rate of T1D among children and adolescents aged 0–14 years has been estimated to be 4.21 per 100,000, with an increase of 2.3% per year from 2007 to 2017 [18]. Parents in China generally adopt authoritative or authoritarian styles to encourage subordination and interdependent behavior among their offspring [19], thereby playing a dominant role in management. In contrast, Caucasian parents tend to adopt authoritative styles, Black parents tend to be more authoritarian, and Hispanic parents often exhibit more permissive styles [20]. Considering the cultural context in China, parental diabetes-specific distress needs to be discussed in depth to support parents in adapting to their caregiver roles. However, there is a lack of studies exploring parental diabetes-specific distress and its associated factors in Chinese parents of children and adolescents with T1D, except for one qualitative study that demonstrated the psychological stress experienced by parents of children and adolescents with T1D [21].

As a psychological phenomenon, parental diabetes-specific distress necessitates the use of accurate and reliable psychological instruments for evaluation, which must account for the developmental differences between children and adolescents. The Problem Areas in Diabetes scales adapted for parents and their children (P-PAID-C) [16] and for parents and their teens (P-PAID-T) [22] have recently been proposed by the American Diabetes Association (ADA) to effectively differentiate diabetes distress–related components of parental care [3]. However, only English, German, and Turkish versions of the scales exist [23, 24].

To enable a culturally appropriate assessment of parental diabetes-specific distress, we conducted cross-cultural adaptation and psychometric validation of the P-PAID-C and P-PAID-T scales, following Brislin's translation model [25]. The adaptation and validation process of the instrument is detailed in the Supporting Information (*Instrument Adaptation and Validation*). The current study aims to compare the differences in parental diabetes-specific distress between parents of children and parents of adolescents and to explore its associated factors. As aforementioned, the aspects of diabetes-specific distress may differ between parents of children with T1D and parents of adolescents with T1D. However, no studies have directly compared the specific aspects or levels of diabetes-specific distress between these two groups. Therefore, it is hypothesized that the levels of personal regimen-specific distress, child regimen-specific distress, negative emotions, and keeping up with chronic demands experienced by the two groups may differ.

## 2. Methods

### 2.1. Participants and Procedure

In this study, a cross-sectional design was adopted, employing a national sample. Data were collected between September 2022 and September 2023. Electronic questionnaires containing a secure recruitment link were delivered via the official accounts on WeChat (one of the most popular social media platforms in China) of the Sinocare Diabetes Foundation, the Second Xiangya Hospital of Central South University, and the First Affiliated Hospital of Xinjiang Medical University (https://mp.weixin.qq.com/s/0vS80laKNl-sFyT9ViZLpQ). These three accounts, with 50,000 followers across the country, provided easy access to T1D patients.

The linked page consisted of four parts: *study profile*, *inclusion and exclusion criteria*, *informed consent*, and a *questionnaire QR*. Considering the ideal division of diabetes management roles across developmental stages [4], this study defined children's ages as 8–12 years and adolescents' ages as 13–18 years. The inclusion criteria were (1) parents of a child/adolescent with T1D; (2) children/adolescents are diagnosed with T1D according to the standard World Health Organization definition and; (3) under insulin therapy for at least 6 months to allow adaptation to living with the disease [26]; and (4) mandarin proficient. Parents were excluded if children/adolescents had serious diabetes complications, including neuropathy, kidney disease, eye disease, or amputation, or serious physical or psychiatric conditions, such as asthma, hypertension, untreated attention deficit hyperactivity disorder, or pervasive developmental disorder.

After conducting a self-check based on the inclusion criteria, participants read and signed an informed consent form that included details about the study's purposes, procedures, and duration. The participants were informed that their anonymity and confidentiality were assured, that their participation was voluntary, and that they could withdraw at any time. They then scanned the QR code to respond to the questionnaires online. Only one parent per child could participate the questionnaire survey. The questionnaire was designed to ensure that every question was completed before submission. The respondents were compensated with 50 yuan (US$7.14) for their participation. Ethical clearance for the study was obtained from the Ethics Committee of the Xiangya School of Nursing, Central South University (E202394).

### 2.2. Measures

Diabetes-specific distress was evaluated using the P-PAID-C and P-PAID-T scales, which are diabetes-specific questionnaires with 16 items and 15 items, respectively. Respondents rate how much each item has bothered them over the previous month using a six-point Likert scale (1 = not a problem, 6 = serious problem), with higher scores indicating greater distress. Summary scores are computed as the sum of the items divided by the number of items answered. The Cronbach's alphas for the Chinese versions of the P-PAID-C and P-PAID-T scales were both 0.97. Both scales included four domains: *negative emotions*, *keeping up with chronic demands*, *personal regimen-specific distress*, and *child regimen-specific distress*. The distress cutoff score of the P-PAID-T scale was determined to be 54 [22], and the cutoff score of the P-PAID-C scale remains unclear. Considering the previous reporting experience from the literature based on population distribution [16], the cutoff score can be deemed high or low if the total score is 1 standard deviation (SD) above or below the mean score.

Perceived stress was evaluated using the Perceived Stress Scale [27]. The Chinese version of the Perceived Stress Scale (CPSS) consists of 14 items and is scored on a five-point Likert scale (0 = never, 4 = very often), with higher scores indicating higher perceived stress levels [28]. The Cronbach's alpha of the CPSS is 0.78, and the cutoff score has been determined to be 26, which indicates excessive stress [28].

Anxiety symptoms were evaluated using the Self-Rating Anxiety Scale (SAS) [29]. The Chinese version consists of 20 items and is rated on a four-point Likert scale (1 = never or occasionally, 4 = most of the time), with higher scores indicating greater anxiety [30]. The Cronbach's alpha of the SAS is 0.89, and the standard score ranges are 25–49 (normal), 50–59 (mild anxiety symptoms), 60–69 (moderate anxiety symptoms), and 70–100 (severe anxiety symptoms) [31].

Depressive symptoms were evaluated using the Self-Rated Depression Scale (SDS) [32]. The Chinese version consists of 20 items and is rated on a four-point Likert scale (1 = never or occasionally, 4 = most of the time), with higher scores indicating more severe depressive symptoms [33]. The Cronbach's alpha of the SDS is 0.89, and the standard score ranges are 25–49 (normal), 50–59 (mild depressive symptoms), 60–69 (moderate depressive symptoms), and 70–100 (severe depressive symptoms) [31].

Parent–child conflict was evaluated using the Family Environment Scale [34]. The Chinese version of the Family Environment Scale (CFES) has two subscales with 16 items [35]. The frequency scale is scored on a five-point Likert scale (1 = never, 5 = several times a day). The intensity scale is also scored on a five-point Likert scale (1 = never, 5 = very intense). Higher scores indicate a higher frequency and intensity of parent–child conflict. The Cronbach's alphas are 0.87 for the frequency scale and 0.89 for the intensity scale [36].

Sociodemographic and T1D-related characteristics were collected using a self-designed questionnaire developed by the research team. The parents' sociodemographic data included relationship to offspring, ethnicity, age, education level, family structure, and annual family income. Parental education levels were categorized as 9 years or less, 10–12 years, and more than 12 years. Family structure was categorized as a two-parent household or a one-parent household. The sociodemographic data of the children and adolescents included age and sex. The T1D-related characteristics included family history of diabetes, diabetes duration, insulin pump therapy, the most recent HbA1c value, and episodes of frequent hypoglycemia and diabetic ketoacidosis (DKA) in the past 6 months. Frequent hypoglycemia was defined as approximately two to four times per week or more [37].

### 2.3. Statistical Analysis

According to the sample-to-variable ratio rule [38], a minimum observation-to-variable ratio of 20:1 was preferred. With 19 predictors, a sample size of 190–380 participants was needed. Considering that 16% of the online questionnaires were invalid [39], the sample size ranges for the parents of children with T1D and the parents of adolescents with T1D in this study were 227–453, respectively.

All statistical analyses were conducted and double-checked by two researchers using SPSS 27.0. Continuous variables are presented as mean and SD. Categorical variables are presented as frequencies and percentages. Since the Kolmogorov–Smirnov tests indicated nonnormality for continuous variables, Wilcoxon rank-sum tests were used for group comparisons. First, Spearman correlation analysis was performed to analyze the correlations among continuous variables, such as parental anxiety symptoms, perceived stress, depressive symptoms, parent–child conflict, and diabetes-specific distress. Second, the Kruskal–Wallis test and Mann–Whitney *U* test were used for univariate analysis of the associations between parental diabetes-specific distress and potential categorical influencing factors. Multiple linear regression models with stepwise methods were then carried out to determine whether parental diabetes-specific distress was associated with the independent variables. Finally, a forced-entry, a priori, three-level, hierarchical linear regression analysis was used to analyze the risk factors of diabetes-specific distress in both parents of children with T1D and parents of adolescents with T1D. Parent demographic variables were obtained in Model 1. Model 2 predicts offspring's demographic and clinical variables based on Model 1. Model 3 predicts psychological symptoms in parents based on Model 2. Bootstrapping with 10,000 resamples was used to generate the accelerated bias and the corrected 95% confidence intervals (95% CI) and associated *p*-values for all inferential tests. Variance inflation factors (VIFs) were used to assess potential multicollinearity among predictors in the full model. Statistical significance was defined as a two-sided *p*-value of less than 0.05.

## 3. Results

### 3.1. Sample Description

In total, 425 parents of children with T1D and 295 parents of adolescents with T1D were enrolled. Sixty parents of children with T1D (14.12%) and 27 parents of adolescents with T1D (9.15%) were excluded because of invalid questionnaires. The analyzed sample included 365 parents of children with T1D (age range 20–60 years; *M* = 37.11, SD = 5.63) and 268 parents of adolescents with T1D (age range 30–56 years; *M* = 41.53, SD = 4.69). For the parents of both children with T1D and adolescents with T1D, most were Han Chinese (96.71%, 353/365, and 95.52%, 256/268, respectively), and approximately half of the respondents were fathers (54.52%, 199/365, and 58.96%, 158/268, respectively). Regarding offspring, 64.11% of the children with T1D (234/365) and 61.57% of the adolescents with T1D (165/268) had a family history of diabetes. Clinically, 87.67% of the children with T1D (320/365) and 91.42% of the adolescents with T1D (245/268) did not use insulin pump therapy. In terms of HbA1c, 60.27% of the children with T1D (220/365) and 62.69% of the adolescents with T1D (168/268) did not meet the HbA1c target (HbA1c < 7.5%) [40]. The sociodemographic and T1D-related characteristics are shown in Tables 1 and 2.

### 3.2. Comparison of Diabetes-Specific Distress Between the Parents of Children With T1D and the Parents of Adolescents With T1D

The mean total score of the P-PAID-C scale was 66.57 (SD = 18.46) with a range from 16–96, and the mean summary score was 4.16 (SD = 1.15). The mean score of the Chinese version of the P-PAID-T scale was 59.68 (SD = 17.52), ranging from 15 to 90, and the mean summary score was 3.97 (SD = 1.17). Notably, 70.15% of the parents of adolescents with T1D (188/268) had higher scores than the cutoff value, and 57.81% (211/365) of the parents of children with T1D reported distress levels of at least 1 SD above the mean of the study sample. The parents of children with T1D had higher levels of diabetes-specific distress than the parents of adolescents with T1D (*p*  < 0.01). Moreover, the parents of children with T1D had higher scores in all domains than the parents of adolescents with T1D (*p*  < 0.05), with the highest mean score reported in the domain of *negative emotions*. The detailed diabetes-specific distress scores of the parents are displayed in Table 3.

### 3.3. Associations Between Sociodemographics, T1D-Related Characteristics, and Diabetes-Specific Distress

Regarding the parents of children with T1D, fathers experienced more diabetes-specific distress than mothers (*p*=0.016). Moreover, higher parental diabetes-specific distress was associated with higher annual family income (*p*=0.024), family history of diabetes (*p*=0.004), children not using an insulin pump (*p*=0.03), not meeting the HbA1c target (*p*=0.007), and more episodes of frequent hypoglycemia (*p*=0.032) and DKA (*p* < 0.001) in the previous 6 months.

Regarding the parents of adolescents with T1D, higher parental diabetes-specific distress was associated with older age of fathers (*p*=0.039) and mothers (*p*=0.040), father's lower education level (*p*=0.018), family history of diabetes (*p*=0.021), adolescents not meeting the HbA1c target (*p*=0.01), and more episodes of frequent hypoglycemia (*p*=0.016) and DKA (*p* < 0.001) in the previous 6 months. Detailed results are shown in Tables 1 and 2.

### 3.4. Correlations Between Anxiety Symptoms, Perceived Stress, Depressive Symptoms, Parent–Child Conflict, HbA1c in Children and Adolescents, and Diabetes-Specific Distress

Parental anxiety symptoms, perceived stress, depressive symptoms, parent–child conflict, and HbA1c in children and adolescents were all positively associated with diabetes-specific distress in the parents of children with T1D and the parents of adolescents with T1D (*p*  < 0.05). Detailed correlations are displayed in Table 4.

### 3.5. Hierarchical Regression Models of Parental Diabetes-Specific Distress

For the parents of children with T1D, in Model 3 (*F* = 8.497, *p*  < 0.001, *R*^2^ = 0.433, adjusted *R*^2^ = 0.382), higher annual family income (≥US$2857), more parental anxiety symptoms and perceived stress, and children's higher frequency of hypoglycemia in the previous 6 months (*β*  =  0.091–0.614, *p* < 0.05) were associated with higher levels of diabetes-specific distress. In addition, fathers with more than 12 years of education (*β*  = −0.141, *p* < 0.05) were associated with lower levels of diabetes-specific distress. The Model 3 results are shown in Table 5.

As for the parents of adolescents with T1D, in Model 3 (*F* = 4.955, *p*  < 0.001, *R*^2^ = 0.385, adjusted *R*^2^ = 0.308), more parental anxiety symptoms and perceived stress (*β*  = 0.137–0.491, *p* < 0.05) were associated with higher levels of diabetes-specific distress. Furthermore, fathers with more than 9 years of education (*β*  = −0.303 to approximately −0.215, *p* < 0.01) were associated with lower levels of diabetes-specific distress. The VIF values for all the included variables were less than 10, implying that there were no signs of multicollinearity. Model 3 results are shown in Table 5.

## 4. Discussion

This study advances the literature by comparing parental diabetes-specific distress between parents of children with T1D and parents of adolescents with T1D at different developmental stages, using validated specific instruments, and further exploring the associated factors within a diverse population sample from 26 provinces in China. Compared to the parents of adolescents with T1D, the parents of children with T1D exhibited higher diabetes-specific distress overall and across all four domains. Higher family incomes and children's episodes of frequent hypoglycemia significantly affected the parents of children with T1D. Fathers with 9 years of education or less, as well as parents with higher levels of anxiety symptoms and perceived stress, were more likely to report higher diabetes-specific distress in both groups.

In this study, 57.81% of parents of children with T1D and 70.15% of parents of adolescents with T1D reported high levels of diabetes-specific distress; however, rates in the United States have been found to be 45% for parents of children with T1D and 60% for parents of adolescents with T1D [9]. Traditional cultural norms in different countries may explain this difference. Compared to Western countries, Chinese parents often feel a greater sense of responsibility and worry about their children facing difficulties in school, finding a job, and securing a spouse. They also experience shame, fear, and guilt stemming from societal stigma [41], which may contribute to higher levels of parental diabetes-specific distress. More specifically, in China, parents of children with T1D had higher levels of diabetes-specific distress than the parents of adolescents with T1D. It is possible that adolescents with T1D possess a certain level of autonomy in managing their illness [4], allowing their parents to gradually diminish their dominant role in the caregiving process and experience fewer worries.

In terms of associated factors, our results surprisingly indicated that, contrary to previous findings in Singapore [13] and the United States [16], higher annual family income was associated with higher diabetes-specific distress in parents of children with T1D. Given the labor market mismatch [42] and intense competition for personnel [43] in China, high-income parents may experience more work-related stress and may lack the emotional reserves or time to adequately care for children with T1D [44]. This can lead to feelings of guilt and worry about their children's condition, resulting in higher parental diabetes-specific distress. Regarding clinical factors, among the parents of children with T1D, a higher frequency of hypoglycemia in the previous 6 months was associated with higher parental diabetes-specific distress. This finding is aligned with a previous systematic review of 1895 parents of children/adolescents with T1D across six countries [45]. The emotional burden of managing frequent hypoglycemia in children with T1D can lead to feelings of guilt, worry, and helplessness [45], leading to higher diabetes-specific distress.

Despite the disparities in the associated factors of diabetes-specific distress between the parents of children with T1D and the parents of adolescents with T1D, there were still some shared factors, such as father's education level, anxiety symptoms, and perceived stress. Consistent with a previous study in Singapore [13], fathers having 9 years or less of education reported higher diabetes-specific distress. Less-educated parents often have less knowledge and social support to manage complex tasks related to their offspring's disease [46]. They may feel overwhelmed by T1D management and may experience higher diabetes-specific distress. Regarding psychological indicators, our study found that parental diabetes-specific distress exhibited a moderate association with perceived stress and a stronger correlation with anxiety symptoms. Previous evidence has shown that parental stress and anxiety symptoms are linked to increased pediatric parenting stress and lower self-efficacy in diabetes care for offspring [47], which may contribute to parental diabetes-specific distress.

Although not statistically significant in the regression model, some factors were noteworthy. We found that fathers experienced higher diabetes-specific distress than mothers in the parents of children with T1D. This finding diverges from previous studies in that mothers experienced more emotional distress [48] and greater feelings of helplessness than fathers [49]. The observed differences may be due to data collection during the stay-home policy during the COVID-19 pandemic, when fathers were more likely to be involved in their children's disease management. As for other clinical factors, consistent with previous studies in the United States, the use of insulin pumps in children was inversely associated with parental diabetes-specific distress [11]. However, in the regression model, this study failed to identify a negative association between insulin pump use and parental diabetes-specific distress. This may be related to the low proportion of insulin pump use in the study population (12.33%). Only 15.21% of children and adolescents with T1D in China are regular users of insulin pumps [50], compared to 67.5% in the United States [11].

Several limitations of this study should also be acknowledged. First, the sample was limited to families who actively engaged with social media, which may impede the generalizability of the findings. Second, the evidence was based on cross-sectional data, including self-reported HbA1c values, which did not allow for the examination of time series revisions. Third, the study focused solely on the psychological profiles of the parents, which could introduce bias, as the psychological factors of their offspring may also affect parental diabetes-specific distress.

This study has both clinical and research implications. First, fathers with 9 years or less of education and parents in high-income families may have more emotional support needs, such as reducing anxiety and perceived stress or helping parents cope with their distress. Second, our findings suggest that education on preventing hypoglycemia may enhance the psychological well-being of parents. Third, future longitudinal studies are needed to establish a causal relationship between sociodemographic and T1D-related characteristics and parental diabetes-specific distress. Finally, investigating whether diabetes-specific distress in children and adolescents with T1D has an interdependent relationship with parental diabetes-specific distress is warranted.

## 5. Conclusion

In China, the parents of children with T1D exhibited higher diabetes-specific distress compared to those of adolescents with T1D, using validated specific instruments. Clinicians should note that high family incomes and children's episodes of frequent hypoglycemia significantly affected the parents of children with T1D and that fathers with 9 years or less of education were particularly affected in both groups. Interventions to reduce anxiety and perceived stress are warranted to reduce parental diabetes-specific distress.

## Figures and Tables

**Table 1:** Sociodemographics of parents of children with type 1 diabetes (T1D) (*n* = 365) and adolescents with T1D (*n* = 268).

Variable	Parents of children with T1D		Parents of adolescents with T1D	
	M (SD) or *n* (%)	*p* ^a^	M (SD) or *n* (%)	*p* ^a^
Relationship to children/adolescents	—	0.016*⁣*^*∗*^	—	0.882
Father	199 (54.52%)	—	158 (58.96%)	—
Mother	166 (45.48%)	—	110 (41.04%)	—
Ethnicity	—	0.125	—	0.111
Han	353 (96.71%)	—	256 (95.52%)	—
Other	12 (3.29%)	—	12 (5.48%)	—
Father's age	37.82 (5.96)	0.719	42.61 (5.52)	0.039*⁣*^*∗*^
≤44	314 (86.03%)	—	173 (64.55%)	—
45–59	49 (13.42%)	—	92 (34.33%)	—
≥60	2 (0.55%)	—	3 (1.12%)	—
Father's education level	—	0.237	—	0.018*⁣*^*∗*^
≤9 years	80 (21.92%)	—	74 (27.61%)	—
10–12 years	62 (16.99%)	—	64 (23.88%)	—
>12 years	223 (61.09%)	—	130 (48.51%)	—
Mother's age	36.35 (5.63)	0.725	40.94 (5.35)	0.040*⁣*^*∗*^
≤44	331 (90.69%)	—	203 (75.75%)	—
45–59	33 (9.04%)	—	63 (23.50%)	—
≥60	1 (0.27%)	—	2 (0.75%)	—
Mother's education level	—	0.352	—	0.347
≤9 years	64 (17.53%)	—	67 (25.00%)	—
10–12 years	86 (23.56%)	—	77 (28.73%)	—
>12 years	215 (58.91%)	—	124 (46.27%)	—
Annual family income (US$)^b^	—	0.024*⁣*^*∗*^	—	0.749
<2857	23 (6.30%)	—	23 (8.58%)	—
2857 to <5714	60 (16.44%)	—	40 (14.93%)	—
5714 to <8571	49 (13.42%)	—	39 (14.55%)	—
8571 to <11,429	52 (14.25%)	—	43 (16.04%)	—
11,429 to <14,286	73 (20.00%)	—	51 (19.03%)	—
≥14,286	108 (29.59%)	—	72 (26.87%)	—

Abbreviations: M, mean; SD, standard deviation.

^a^Kruskal–Wallis test, Mann–Whitney *U* test.

^b^1 dollar ≈7 yuan; the annual family incomes in Chinese yuan are <20,000, 20,000–40,000, 40,000–60,000, 60,000–80,000, 80,000–100,000, and ≥100,000.

*⁣* ^*∗*^ *p*  < 0.05.

**Table 2:** Sociodemographic and type 1 diabetes (T1D)–related characteristics of children with T1D (*n* = 365) and adolescents with T1D (*n* = 268).

Variable	Children with T1D		Adolescents with T1D	
	M (SD) or *n* (%)	*p* ^a^	M (SD) or *n* (%)	*p* ^a^
Age (years)	9.84 (1.46)	—	15.41 (1.69)	—
Sex	—	0.901	—	0.566
Male	252 (69.04%)	—	190 (70.90%)	—
Female	113 (31.96%)	—	78 (29.10%)	—
Family history of diabetes	234 (64.11%)	0.004*⁣*^*∗∗*^	165 (61.57%)	0.021*⁣*^*∗*^
Diabetes duration	—	0.417	—	0.409
6 months to <1 year	22 (6.03%)	—	47 (17.54%)	—
1 year to <5 years	297 (81.37%)	—	165 (61.57%)	—
≥5 years	46 (12.60%)	—	56 (20.89%)	—
Insulin pump therapy	—	0.030*⁣*^*∗*^	—	0.097
Yes	45 (12.33%)	—	23 (8.58%)	—
No	320 (87.67%)	—	245 (91.42%)	—
HbA1c	8.43 (2.63)	0.007*⁣*^*∗∗*^	8.51 (2.68)	0.010*⁣*^*∗*^
Meet the target	145 (39.73%)	—	100 (37.31%)	—
Do not meet the target	220 (60.27%)	—	168 (62.69%)	—
Metabolic complications, past 6 months	—	—	—	—
Frequent hypoglycemia	310 (84.93%)	0.032*⁣*^*∗*^	230 (85.82%)	0.016*⁣*^*∗*^
DKA	189 (51.78%)	<0.001*⁣*^*∗∗∗*^	137 (51.12%)	0.001*⁣*^*∗∗*^

Abbreviations: M, mean; SD, standard deviation.

^a^Kruskal–Wallis test, Mann–Whitney *U* test.

*⁣* ^*∗*^ *p*  < 0.05.

*⁣* ^*∗∗*^ *p*  < 0.01.

*⁣* ^*∗∗∗*^ *p*  < 0.001.

**Table 3:** Diabetes-specific distress scores between parents of children with type 1 diabetes (T1D) and parents of adolescents with T1D (mean ± SD).

Parental diabetes-specific distress^a^	Parents of children with T1D	Parents of adolescents with T1D	*Z*	*p* ^b^
Total score	66.57 ± 18.46	59.68 ± 17.52	—	—
Summary score	4.16 ± 1.15	3.97 ± 1.17	−2.605	0.009*⁣*^*∗∗*^
Personal regimen-specific distress	4.11 ± 1.25	3.92 ± 1.27	−2.496	0.013*⁣*^*∗*^
Child regimen-specific distress	4.00 ± 1.26	3.80 ± 1.27	−2.857	0.004*⁣*^*∗∗*^
Negative emotions	4.32 ± 1.20	4.13 ± 1.19	−2.647	0.008*⁣*^*∗∗*^
Keeping up with chronic demands	4.13 ± 1.30	3.96 ± 1.28	−2.083	0.037*⁣*^*∗*^

Abbreviation: SD, standard deviation.

^a^Measured by the P-PAID-C scale for parents of children with T1D and the P-PAID-T scale for parents of adolescents with T1D.

^b^Mann–Whitney *U* test.

*⁣* ^*∗*^ *p*  < 0.05.

*⁣* ^*∗∗*^ *p*  < 0.01.

**Table 4:** Intercorrelations for study variables.

Variable	Parental diabetes-specific distress			
	Parents of children with T1D		Parents of adolescents with T1D	
	M (SD) or *n* (%)	*r* ^a^	M (SD) or *n* (%)	*r* ^a^
Anxiety symptoms	58.95 (13.54)	0.592*⁣*^*∗∗∗*^	57.81 (14.52)	0.463*⁣*^*∗∗∗*^
Normal	84 (23.01%)	—	73 (27.24%)	—
Mild	74 (20.27%)	—	43 (16.04%)	—
Moderate	122 (33.43%)	—	98 (36.57%)	—
Severe	85 (23.29%)	—	54 (20.15%)	—
Perceived stress	28.27 (5.05)	0.26*⁣*^*∗∗∗*^	28.29 (5.65)	0.194*⁣*^*∗∗*^
Excessive stress	307 (84.11%)	—	229 (85.45%)	—
Depressive symptoms	59.42 (9.66)	0.304*⁣*^*∗∗∗*^	58.66 (10.21)	0.207*⁣*^*∗∗∗*^
Normal	55 (15.07%)	—	43 (16.04%)	—
Mild	55 (15.07%)	—	54 (20.15%)	—
Moderate	225 (61.64%)	—	150 (55.97%)	—
Severe	30 (8.22%)	—	21 (7.84%)	—
Parent–child conflict	41.05 (16.04)	0.285*⁣*^*∗∗∗*^	39.35 (17.04)	0.233*⁣*^*∗∗∗*^
Children/adolescents' HbA1c	8.43 (2.63)	0.132*⁣*^*∗*^	8.51 (2.68)	0.169*⁣*^*∗∗*^

Abbreviations: M, mean; SD, standard deviation; T1D, type 1 diabetes.

^a^Spearman's correlation coefficient.

*⁣* ^*∗*^ *p*  < 0.05.

*⁣* ^*∗∗*^ *p*  < 0.01.

*⁣* ^*∗∗∗*^ *p*  < 0.001.

**Table 5:** Hierarchical regression analysis for diabetes-specific distress in parents of children with type 1 diabetes (T1D) (*n* = 365) and parents of adolescents with T1D (*n* = 268).

Variable	Diabetes-specific distress in parents of children with T1D		Diabetes-specific distress in parents of adolescents with T1D	
	*β* (95 % CI)	*p*	*β* (95 % CI)	*p*
Parental characteristics	—	—	—	—
Father's education level	—	—	—	—
≤9 years	Reference	—	—	—
10–12 years	0.023 (−4.232; 6.492)	0.679	−0.215 (−14.572; −3.080)	0.003*⁣*^*∗∗*^
>12 years	−0.141 (−10.139; −0.508)	0.030*⁣*^*∗*^	−0.303 (−16.365; −4.851)	<0.001*⁣*^*∗∗∗*^
Annual family income (US$)	—	—	—	—
<2857	Reference	—	—	—
2857 to < 5714	0.162 (0.724; 15.347)	0.031*⁣*^*∗*^	—	—
5714 to < 8571	0.043 (−5.169; 9.788)	0.544	—	—
8571 to <11,429	0.171 (1.490; 16.536)	0.019*⁣*^*∗*^	—	—
11,429 to < 14,286	0.189 (1.654; 15.773)	0.016*⁣*^*∗*^	—	—
≥14,286	—	—	—	—
Anxiety symptoms	—	—	—	—
Normal	Reference	—	—	—
Mild	0.177 (2.388; 13.802)	0.006*⁣*^*∗∗*^	0.135 (−0.656; 13.520)	0.075
Moderate	0.386 (9.407; 20.762)	<0.001*⁣*^*∗∗∗*^	0.220 (1.122; 14.884)	0.023*⁣*^*∗*^
Severe	0.614 (20.475; 33.069)	<0.001*⁣*^*∗∗∗*^	0.491 (14.053; 28.785)	<0.001*⁣*^*∗∗∗*^
Perceived stress	—	—	—	—
Normal	Reference	—	—	—
Excessive stress	0.101 (0.454; 9.694)	0.031*⁣*^*∗*^	0.190 (3.817; 15.010)	0.001*⁣*^*∗∗*^
Children/adolescents' characteristics	—	—	—	—
Frequent hypoglycemia, past 6 months	—	—	—	—
No	Reference	—	—	—
Yes	0.091 (0.120; 9.310)	0.044*⁣*^*∗*^	—	—

Abbreviation: *β*, standardized coefficients.

*⁣* ^*∗*^ *p*  < 0.05.

*⁣* ^*∗∗*^ *p*  < 0.01.

*⁣* ^*∗∗∗*^ *p*  < 0.001.

## Data Availability

The data supporting the findings of this study are available from the corresponding author (Jia Guo) upon reasonable request.

## Supporting Information

Supporting Information The adaptation and validation process of the instrument is detailed in the Supporting Information (*Instrument Adaptation and Validation*).

## References

[1] Barnett R. (2018). Type 1 Diabetes. *The Lancet*.

[2] International Diabetes Federation (2025). *IDF Diabetes Atlas 2025 | IDF Diabetes Atlas*.

[3] ElSayed N. A., Aleppo G., Aroda V. R. (2023). 14. Children and Adolescents: *Standards of Care in Diabetes—2023*. *Diabetes Care*.

[4] Markowitz J. T., Garvey K. C., Laffel L. M. B. (2015). Developmental Changes in the Roles of Patients and Families in Type 1 Diabetes Management. *Current Diabetes Reviews*.

[5] Wentzell K., Volkening L., Laffel L. (2022). Concordance and Discordance Between Youth and Their Parents’ Reports of Diabetes Distress: The Importance of Youth Perceptions. *Journal of Diabetes and its Complications*.

[6] Law G. U., Walsh J., Queralt V., Nouwen A. (2013). Adolescent and Parent Diabetes Distress in Type 1 Diabetes: The Role of Self-Efficacy, Perceived Consequences, Family Responsibility and Adolescent–Parent Discrepancies. *Journal of Psychosomatic Research*.

[7] Lv W., Luo J., Long Q., Yang J., Wang X., Guo J. (2021). Factors Associated With Adherence to Self-Monitoring of Blood Glucose Among Young People With Type 1 Diabetes in China: A Cross-Sectional Study, Patient Prefer. *Patient Preference and Adherence*.

[8] Whittemore R., Jaser S., Chao A., Jang M., Grey M. (2012). Psychological Experience of Parents of Children With Type 1 Diabetes. *The Diabetes Educator*.

[9] Evans M., Ellis D. A., Vesco A. T. (2024). Diabetes Distress in Urban Black Youth With Type 1 Diabetes and Their Caregivers: Associations With Glycemic Control, Depression, and Health Behaviors. *Journal of Pediatric Psychology*.

[10] Skinner T. C., Joensen L., Parkin T. (2020). Twenty-Five Years of Diabetes Distress Research. *Diabetic Medicine*.

[11] Patton S. R., Kahhan N., Pierce J. S., Benson M., Fox L. A., Clements M. A. (2023). Parental Diabetes Distress is a Stronger Predictor of Child HbA1c Than Diabetes Device use in School-Age Children With Type 1 Diabetes. *BMJ Open Diabetes Research & Care*.

[12] Healey D., Gray A. R., Chae M. (2018). The Role of Parent and Child Self-Regulation in Children’s Glycemic Control. *Health Psychology*.

[13] Toh Z. Q., Koh S. S. L., Lim P. K., Lim J. S. T., Tam W., Shorey S. (2021). Diabetes-Related Emotional Distress Among Children/Adolescents and Their Parents: A Descriptive Cross-Sectional Study. *Clinical Nursing Research*.

[14] Hessler D., Fisher L., Polonsky W., Johnson N. (2016). Understanding the Areas and Correlates of Diabetes-Related Distress in Parents of Teens With Type 1 Diabetes. *Journal of Pediatric Psychology*.

[15] Inverso H., Moore H. R., Morrow T., Jaser S. S., Streisand R. (2021). 906-P: Parents Experience Diabetes Distress Too: Parental Reports and Association With Quality of Life and Teen A1C. *Diabetes*.

[16] Evans M. A., Weil L. E. G., Shapiro J. B. (2019). Psychometric Properties of the Parent and Child Problem Areas in Diabetes Measures. *Journal of Pediatric Psychology*.

[17] Hilliard M. E., Holmes C. S., Chen R., Maher K., Robinson E., Streisand R. (2013). Disentangling the Roles of Parental Monitoring and Family Conflict in Adolescents’ Management of Type 1 diabetes.. *Health Psychology*.

[18] Liu C., Yuan Y. C., Guo M. N. (2021). Incidence of Type 1 Diabetes May Be Underestimated in the Chinese Population: Evidence From 21.7 Million People Between 2007 and 2017. *Diabetes Care*.

[19] Xu Y., Farver J. A. M., Zhang Z., Zeng Q., Yu L., Cai B. (2005). Mainland Chinese Parenting Styles and Parent–Child Interaction. *International Journal of Behavioral Development*.

[20] Spera C. (2005). A Review of the Relationship Among Parenting Practices, Parenting Styles, and Adolescent School Achievement. *Educational Psychology Review*.

[21] Tong H., Qiu F., Fan L. (2022). Characterising Common Challenges Faced by Parental Caregivers of Children With Type 1 Diabetes Mellitus in Mainland China: A Qualitative Study. *BMJ Open*.

[22] Shapiro J. B., Vesco A. T., Weil L. E. G., Evans M. A., Hood K. K., Weissberg-Benchell J. (2018). Psychometric Properties of the Problem Areas in Diabetes: Teen and Parent of Teen Versions. *Journal of Pediatric Psychology*.

[23] Saßmann H., Kim-Dorner S. J., Framme J. (2023). Psychometric Properties of the German Teen and Parent Versions of the Problem Areas in Diabetes Scale (PAID). *Psychological Assessment*.

[24] Sari S. A., Agadayi E., Celik N., Karahan S., Tan A. Komurluoglu, Doger E. (2023). The Turkish Version of the Problem Areas in Diabetes-Parents of Teens (P-PAID-T): Cross-Cultural Adaptation, Reliability, and Validity. *Journal of Pediatric Nursing*.

[25] Jones P. S., Lee J. W., Phillips L. R., Zhang X. E., Jaceldo K. B. (2001). An Adaptation of Brislin???s Translation Model for Cross-Cultural Research. *Nursing Research*.

[26] McGill D. E., Volkening L. K., Pober D. M., Muir A. B., Young-Hyman D. L., Laffel L. M. (2018). Depressive Symptoms at Critical Times in Youth With Type 1 Diabetes: Following Type 1 Diabetes Diagnosis and Insulin Pump Initiation. *Journal of Adolescent Health*.

[27] Cohen S., Kamarck T., Mermelstein R. (1983). A Global Measure of Perceived Stress. *Journal of Health and Social Behavior*.

[28] Yang T-Z., Huang H-T. (2003). [An Epidemiological Study on Stress Among Urban Residents in Social Transition Period]. *Zhonghua Liu Xing Bing Xue Za Zhi*.

[29] Zung W. W. (1971). A Rating Instrument For Anxiety Disorders. *Psychosomatics*.

[30] Wang Z. Y., Chi Y. F. (1984). Chinese Version of Zung’ s Self-Rating Anxiety Scale. *Shanghai Archives of Psychiatry*.

[31] Quan D. (2012). Differential Validity of SAS and SDS Among Psychiatric Non-Psychotic Outpatients and Their Partners. *Chinese Mental Health Journal*.

[32] Zung W. W. (1965). A Self-Rating Depression Scale. *Archives of General Psychiatry*.

[33] Wang Z. Y., Chi Y. F. (1984). Chinese Version of Zung’s Self-Rating Depression Scale. *Shanghai Archives of Psychiatry*.

[34] Moos R. H., Moos B. S. (1976). A Typology of Family Social Environments. *Family Process*.

[35] Fang X. Y., Dong Q. (1998). Parent-Child Conflict in First and Second Middle School Students. *Journal of Psychological Science*.

[36] Diao J., Sang B. (2009). Research on Family Members’ Cognition Difference of Parent-Child Conflict in Children’s Puberty. *Contemporary Young Research*.

[37] Allen C., LeCaire T., Palta M. (2001). Risk Factors for Frequent and Severe Hypoglycemia in Type 1 Diabetes. *Diabetes Care*.

[38] Memon M. A., Ting H., Cheah J.-H., Thurasamy R., Chuah F., Cham T. H. (2020). Sample Size for Survey Research: Review and Recommendations. *Journal of Applied Structural Equation Modeling*.

[39] Xiong H. Y., Zhang G., Wang L. (2022). Psychological Research of the Children With Chronic Kidney Disease and Their Guardians During the COVID-19 Pandemic. *Frontiers in Public Health*.

[40] Chinese Diabetes Society, Chinese Endocrinologist Association, Chinese Society of Endocrinology, Chinese Pediatric Society, Zhiguang Z. (2022). Guidelines for the Diagnosis and Treatment of Type 1 Diabetes Mellitus in China (2021 Edition). *Chinese Journal of Diabetes Mellitus*.

[41] Jaacks L. M., Liu W., Ji L., Mayer-Davis E. J. (2015). Type 1 Diabetes Stigma in China: A Call to End the Devaluation of Individuals Living With a Manageable Chronic Disease. *Diabetes Research and Clinical Practice*.

[42] (2025). China Faces Structural Mismatch in Labour Market as Interest in Manufacturing Falls, Amid Record High Youth Unemployment. https://www.channelnewsasia.com/asia/china-faces-structural-mismatch-labour-market-interest-manufacturing-falls-amid-record-high-youth-unemployment-3633786.

[43] Boland B., Dong K., Blanchette J., Hass R., Ye E. (2024). How China’s Human Capital Impacts Its National Competitiveness. https://www.csis.org/analysis/how-chinas-human-capital-impacts-its-national-competitiveness.

[44] Helgeson V. S., Becker D., Escobar O., Siminerio L. (2012). Families With Children With Diabetes: Implications of Parent Stress for Parent and Child Health. *Journal of Pediatric Psychology*.

[45] Jensen M. V., Broadley M., Speight J. (2022). the Impact of Hypoglycaemia in Children and Adolescents With Type 1 Diabetes on Parental Quality of Life and Related Outcomes: A Systematic Review. *Pediatric Diabetes*.

[46] K. Rechenberg, R. Whittemore, M. Grey, S. Jaser, the TeenCOPE Research Group (2016). Contribution of Income to Self-Management and Health Outcomes in Pediatric Type 1 Diabetes. *Pediatric Diabetes*.

[47] Streisand R., Mackey E. R., Elliot B. M. (2008). Parental Anxiety and Depression Associated With Caring for a Child Newly Diagnosed With Type 1 Diabetes: Opportunities for Education and Counseling. *Patient Education and Counseling*.

[48] Haugstvedt A., Wentzel-Larsen T., Rokne B., Graue M. (2011). Perceived Family Burden and Emotional Distress: Similarities and Differences Between Mothers and Fathers of Children With Type 1 Diabetes in a Population-Based Study. *Pediatric Diabetes*.

[49] Majidi S., O’Donnell H., Benson K., Driscoll K. A. (2018). Clinic-Wide Screening of Fear of Hypoglycemia in Mothers and Fathers of Children With Type 1 Diabetes. *Diabetes*.

[50] Hou L., Li X., Liu L. (2021). A Multicenter Survey of Type I Diabetes Mellitus in Chinese Children. *Frontiers in Endocrinology*.

