# Supplementary material for: Parents of Children With Type 1 Diabetes Experienced More Parent-Specific Distress Than Parents of Adolescents in China
Source: Pediatr Diabetes. 2025 Jun 20;2025:5210513. doi: 10.1155/pedi/5210513 (PMC12204744; doi:10.1155/pedi/5210513)
Supplement: Supporting Information — The adaptation and validation process of the instrument is detailed in the Supporting Information (Instrument Adaptation and Validation). [file 5210513.f1.docx]

**Instrument Adaptation and Validation**

Participants were recruited online from March and May 2022 by distributing questionnaire QR code of an online platform (“Survey Star”, Changsha Ran Xing Science and Technology, Changsha, China). The questionnaires were handed out in the official account of Sinocare Diabetes Foundation (https://mp.weixin.qq.com/s/0vS80laKNl-sFyT9ViZLpQ). Inclusion criteria consisted of: (1) parents of a child/adolescent with T1D; (2) children/adolescents are diagnosed with T1D according to the standard World Health Organization definition and; (3) under insulin therapy for at least six months to allow adaptation to living with the disease; (4) mandarin proficient. Parents were excluded if children/adolescents had serious diabetes complications, including neuropathy, kidney disease, eye disease, or amputation, or serious physical or psychiatric conditions, such as asthma, hypertension, untreated attention deficit hyperactivity disorder, or pervasive developmental disorder. The analyzed sample consisted of 215 parents of children with T1D at the age range of 25-60 (M = 36.96, SD = 5.68), and 206 parents of adolescents at the age range of 30-75 (M = 41.07, SD = 5.57).

**Reliability**

The Chinese versions of P-PAID-C and P-PAID-T scales were shown to have good internal consistency. The Cronbach’s alphas for the Chinese versions of the P-PAID-C and P-PAID-T scales were both 0.97. The Pearson coefficients, which were used to illustrate the split-half reliability of scales, were 0.949 and 0.954, respectively. The corrected item–total correlation coefficients for the Chinese versions of the P-PAID-C and P-PAID-T scales ranged from 0.748 to 0.837 and from 0.742 to 0.841, respectively.

**Validity**

SPSS AMOS 27.0 was used for Confirmatory factor analyses (CFA). CFA provides information including chi square by χ^2^/*df*, goodness-of-fit index (GFI), comparative fit index (CFI), Tucker-Lewis Index (TLI), root mean square error of approximation (RMSEA), and root mean square residual (RMR). Values of 1≤ χ^2^/*df* ≤ 5, GFI > 0.90, CFI > 0.90, TLI > 0.90, RMSEA ≤ 0.10, and RMR < 0.10 are considered acceptable. Fit was adequate for the four-factor of Chinese version of P-PAID-C (**Fig. 1**) and P-PAID-T (**Fig. 2)**. The results of CFA of P-PAID-C are as follows: χ^2^/df = 3.874, p < 0.01; RMSR = 0.076; RMSEA = 0.083; GFI = 0.892; TLI = 0.940; CFI = 0.951. The results of CFA of P-PAID-T are as follows: χ^2^/df = 3.331, p < 0.01; RMSR = 0.063; RMSEA = 0.092; GFI = 0.892; TLI = 0.935; CFI = 0.948.

In terms of critical ratio, it was shown that the differences in the P-PAID-C and P-PAID-T scales’ total scores between the high (top 27%) and low (bottom 27%) groups were statistically significant (p < 0.01). Both scales included four domains: *personal regimen-specific distress*, *child regimen-specific distress*, *negative emotions*, and *keeping up with chronic demands* (**[Table 1](https://www.sciencedirect.com/science/article/pii/S016503272200218X?via=ihub#t0005)**).


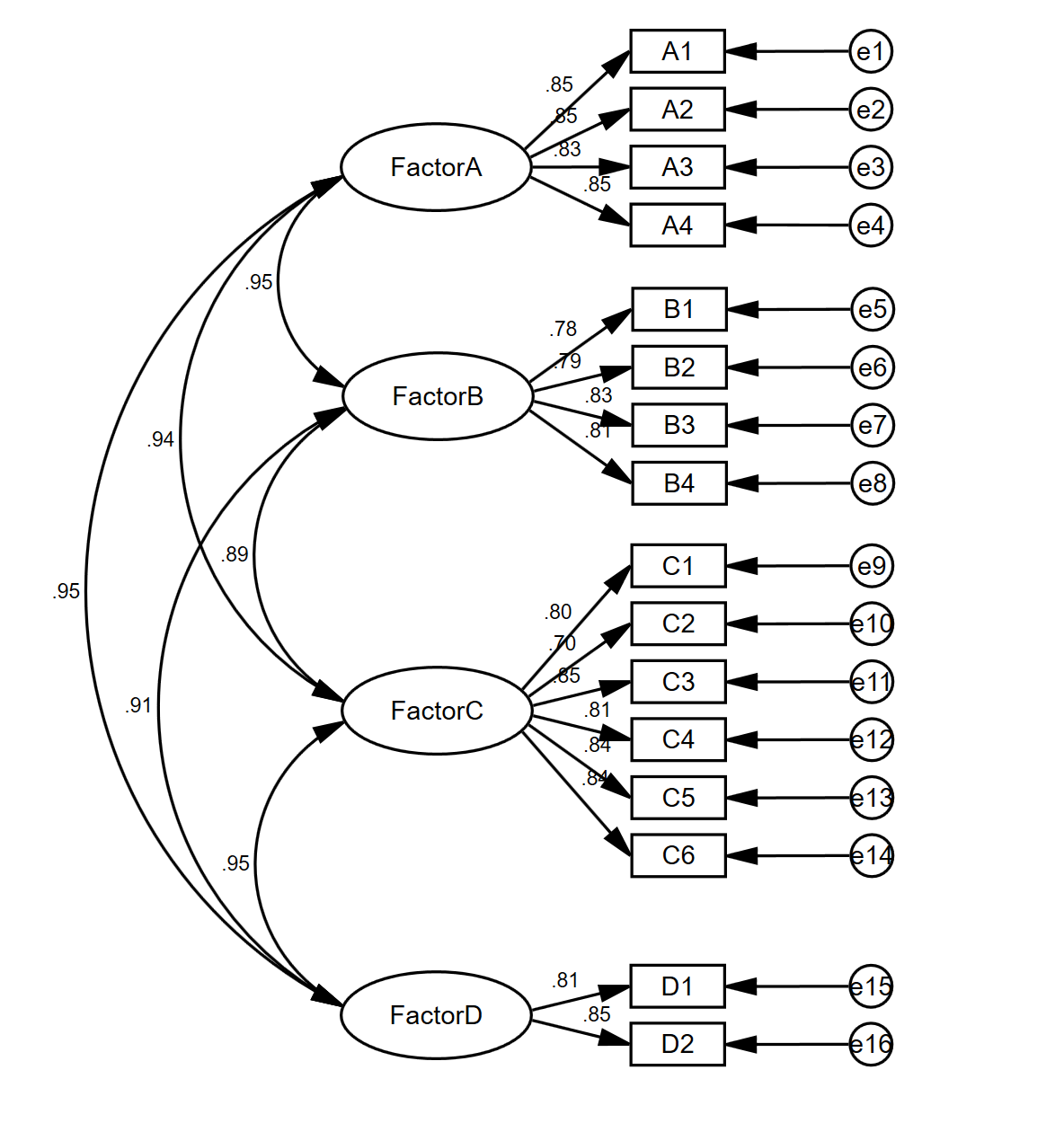


**Fig. 1.** Four-factor model of the Chinese version of P-PAID-C.

Factor A, *personal regimen-specific distress*; Factor B, *child regimen-specific distress*; Factor C, *negative emotions*; Factor D, *keeping up with chronic demands*.


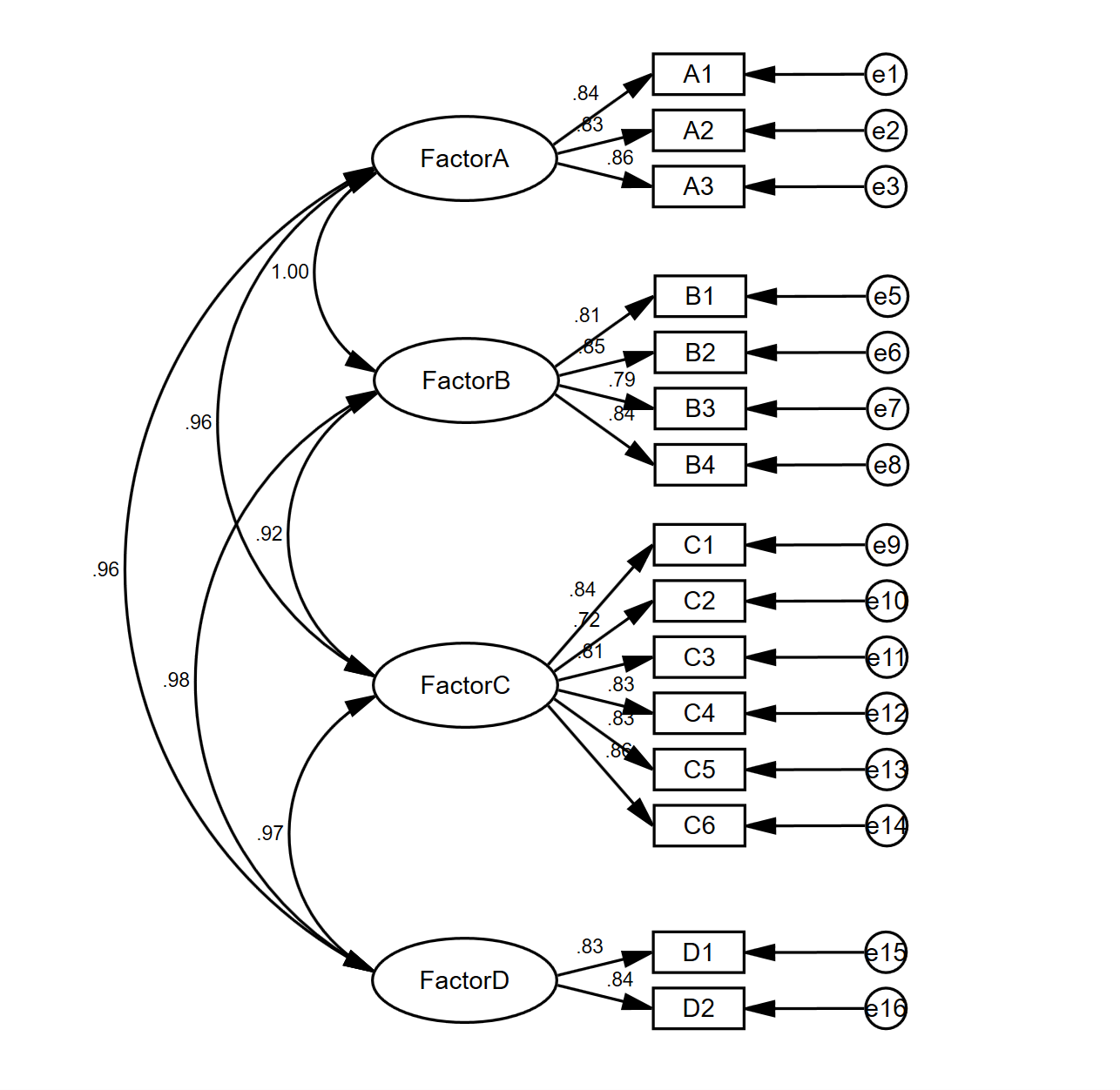


**Fig. 2.** Four-factor model of the Chinese version of P-PAID-T.

Factor A, *personal regimen-specific distress*; Factor B, *child regimen-specific distress*; Factor C, *negative emotions*; Factor D, *keeping up with chronic demands*.

**Table 1.** The specific factors of P-PAID-C and P-PAID-T.

| Factor | | Item ^a^ of P-PAID-C | Item ^a^ of P-PAID-T |
| --- | --- | --- | --- |
| Factor A  personal regimen-specific distress | A1 | High numbers | High numbers |
|  | A2 | Perfect in management | Blood sugars swinging |
|  | A3 | Blood sugars swinging | Failing at regimen |
|  | A4 | Failing at regimen |  |
| Factor B  child regimen-specific distress | B1 | Not checking enough | Not checking enough |
|  | B2 | Friends/family as “diabetes police” | Friends/family as “diabetes police” |
|  | B3 | Parent mistrust | Parent mistrust |
|  | B4 | Missing checks | Missing checks |
| Factor C  negative emotions | C1 | Sad | Sad |
|  | C2 | Angry | Angry |
|  | C3 | Food/eating | Food/eating |
|  | C4 | Complications | Complications |
|  | C5 | Management “off track” | Management “off track” |
|  | C6 | Parent worry about complications | Parent worry about complications |
| Factor D  keeping up with chronic demands | D1 | Overwhelmed | Overwhelmed |
|  | D2 | Tired of diabetes | Tired of diabetes |

Note: P-PAID-C = Problem Area in Diabetes-parent of children version; P-PAID-T = Problem Area in Diabetes-parent of teens version.

^a^ General content indicated and not full item wording.
